# Initial Data on the Molecular Epidemiology of Cryptosporidiosis in Lebanon

**DOI:** 10.1371/journal.pone.0125129

**Published:** 2015-05-07

**Authors:** Marwan Osman, Dima El Safadi, Sadia Benamrouz, Karine Guyot, Eduardo Dei-Cas, El Moukhtar Aliouat, Colette Creusy, Hassan Mallat, Monzer Hamze, Fouad Dabboussi, Eric Viscogliosi, Gabriela Certad

**Affiliations:** 1 Institut Pasteur de Lille, Centre d’Infection et d’Immunité de Lille (CIIL), UMR CNRS 8204, Inserm U1019, Université Lille Nord de France, Biologie et Diversité des Pathogènes Eucaryotes Emergents (BDPEE), Lille, France; 2 Centre AZM pour la recherche en biotechnologies et ses applications, Université Libanaise, Laboratoire de Microbiologie Santé et Environnement, Tripoli, Liban; 3 Faculté Libre des Sciences et Technologies de Lille, Université Catholique de Lille, Université Lille Nord de France, Laboratoire Ecologie et Biodiversité, Lille, France; 4 Centre Hospitalier Régional et Universitaire de Lille & Faculté de Médicine de Lille, Université Lille Nord de France, Laboratoire de Parasitologie-Mycologie, Centre de Biologie et Pathologie, Lille, France; 5 Faculté des sciences pharmaceutiques et biologiques, Université Lille Nord de France, Département de Parasitologie—Mycologie, Lille, France; 6 Service d’Anatomie et de Cytologie Pathologiques, Groupe Hospitalier de l’Université Catholique de Lille, 59020 Lille, France; California Department of Public Health, UNITED STATES

## Abstract

*Cryptosporidium* spp. represent a major public health problem worldwide and infect the gastrointestinal tract of both immunocompetent and immunocompromised persons. The prevalence of these parasites varies by geographic region, and no data are currently available in Lebanon. To promote an understanding of the epidemiology of cryptosporidiosisin this country, the main aim of this study was to determine the prevalence *Cryptosporidium* in symptomatic hospitalized patients, and to analyze the genetic diversity of the corresponding isolates. Fecal specimens were collected in four hospitals in North Lebanon from 163 patients (77 males and 86 females, ranging in age from 1 to 88 years, with a mean age of 22 years) presenting gastrointestinal disorders during the period July to December 2013. The overall prevalence of *Cryptosporidium* spp. infection obtained by modified Ziehl-Neelsen staining and/or nested PCR was 11%, and children <5 years old showed a higher rate of *Cryptosporidium* spp. The PCR products of the 15 positive samples were successfully sequenced. Among them, 10 isolates (66.7%) were identified as *C*. *hominis*, while the remaining 5 (33.3%) were identified as *C*. *parvum*. After analysis of the gp60 locus, *C*. *hominis* IdA19, a rare subtype, was found to be predominant. Two *C*. *parvum* subtypes were found: IIaA15G1R1 and IIaA15G2R1. The molecular characterization of *Cryptosporidium* isolates is an important step in improving our understanding of the epidemiology and transmission of the infection.

## Introduction

*Cryptosporidium* is a protozoan parasite of humans and animals with worldwide distribution. This Apicomplexa is a well-described cause of diarrhea, and is recognized as one of the predominant causes of foodborne and waterborne diseases [1,2]. In immunocompetent individuals, cryptosporidiosis may be symptomatic or asymptomatic. In the first case, the most common symptomatology is acute watery diarrhea, lasting up to 2 weeks after exposure to the parasite (7 days on average). Recovery from diarrhea occurs spontaneously in around ten days. Other symptoms, such as abdominal pain, nausea, vomiting, dehydration, asthenia and weight loss, may be present [3]. In immunocompromised individuals, especially in AIDS patients, *Cryptosporidium* oocyst shedding is persistent, and diarrhea becomes chronic and potentially fatal [4].

Due to the ability of *Cryptosporidium* oocysts to resist conventional water treatment methods and cause waterborne outbreaks, the World Health Organization (WHO) has included this fecally/orally transmitted parasite as a reference pathogen in the design and implementation of the WHO guidelines for drinking water quality. Monitoring for oocysts in water is part of the surveillance to support water safety plans [5,6].

The prevalence of cryptosporidiosis reported in developing countries is 2 to 15 times higher than in industrialized countries [7]. This variation can be attributed to better hygiene among inhabitants, and the prevention of contamination of food and water by oocysts in developed countries.

Nevertheless, the transmission routes in the epidemiology of cryptosporidiosis are not yet entirely clarified, largely due to the fact that traditional diagnostic tools do not allow the identification of sources of parasites, and epidemiologic investigations are expensive to conduct [8]. However, the number of investigations based on the molecular epidemiology of *Cryptosporidium* has increased in the last decade, especially in developing countries, contributing to a better understanding of this public health problem [9]. In particular, information about the situation and impact of cryptosporidiosis in Lebanon is limited, even though other parasitic infections are prevalent [10].

Cryptosporidiosis occurrence rates vary in Middle Eastern countries. Previous reports based on molecular epidemiology among hospitalized patients have shown differing prevalence as follows: 10% in children and adults in Yemen [11], 19% in children in Jordan [12] or 49% in children in Egypt [13]. *C*. *parvum* is the predominant species in this geographic region. Despite the high number of subtypes and allele families of *C*. *parvum* described in these countries, most of the isolates reported belong to two subtype families, IIa and IId [11,12,14,15,16]. Similarly, several subtypes of *C*. *hominis* have been reported, with predominance of the subtype families Ib and Id [12,14,15,16]. The anthroponotic family of *C*. *parvum*, IIc, was also found in this region, but in low proportion [12,14,15]. In particular, two previous epidemiologic surveys among Lebanese HIV patients based on microscopy observation described a cryptosporidiosis rate of between 3.1% and 50% [17,18].

In order to better understand the epidemiology of cryptosporidiosis in Lebanon, the main aim of this study was to determine the prevalence of *Cryptosporidium* in symptomatic hospitalized patients, and to analyze the genetic diversity of the isolates. For the first time at the molecular level, we characterized the species and subtypes of *Cryptosporidium* circulating in Lebanon, a tourist-oriented Middle Eastern country that is a crossroads of the Mediterranean Basin and the Arab hinterland.

## Material and Methods

### Ethics Statement

Authorization to conduct this study was obtained from the Lebanese Minister of Public Health (reference number: 4–39716). The institutional directory review boards of Nini and Al-Shifa’ Hospitals in Tripoli, and Al-Youssef and Rahal Hospitals in Akkar, also approved the protocol of this project, in agreement with Lebanese legislation. Oral and written informed consents were obtained from the parents or legal guardians of the children, or directly in the case of adult patients, after a clear explanation of the research objectives. The present study was conducted in accordance with the Code of Ethics of the World Medical Association (Declaration of Helsinki).

### Sample collection

**Fig 1:**
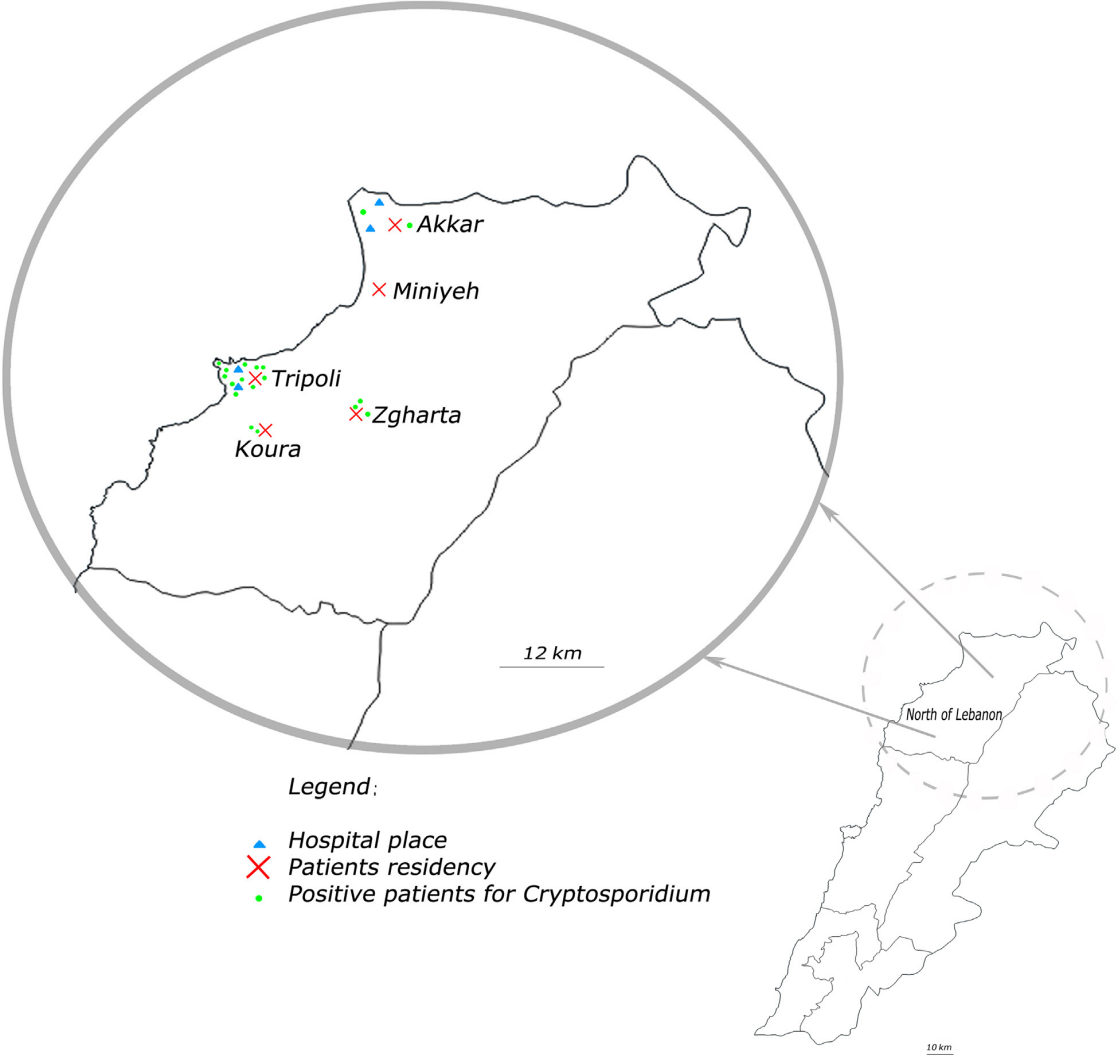
Geographic distribution of cryptosporidiosis cases identified in this study in Lebanon.

This study was conducted in the North Governorate of Lebanon (Fig 1). Fecal specimens were collected in four hospitals in North Lebanon (Nini Hospital and Al-Shifa’ Hospital in Tripoli; Al-Youssef Hospital and Rahal Hospital in Akkar) from 163 hospitalized patients (77 males and 86 females, ranging in age from 1 to 88 years, with a mean age of 22 years) presenting gastrointestinal disorders during the period July to December 2013 (Table 1). All patients were HIV (Human Immunodeficiency Virus) negative. One fecal sample per patient was collected in a sterile container and transported immediately to the Department of Microbiology of the AZM Center in Tripoli.

**Table 1:** Clinical data and *Cryptosporidium* spp. and genotypes among symptomatic patients in Lebanon.

Patient identification	Sex	Age (years)	Hospital (residency)	Symptoms	MZN staining	*Cryptosporidium* species (18S rRNA)	Locus gp60	Co-infection
MH1	F	60	Al-Shifa’ (Tripoli)	AP/D	+	*C*. *parvum*	IIaA15G2R1	-
MH2	M	15	Al-Shifa’ (Tripoli)	AP/D/F	+	*C*. *parvum*	IIaA15G1R1	-
MH3	F	76	Nini (Tripoli)	AP/D/V	+	*C*. *parvum*	IIaA15G1R1	-
MH4	F	32	Nini (Zgharta)	AP/D	+	*C*. *parvum*	IIaA15G1R1	*E*. *hartmanni*
MH5	F	1	Nini (Tripoli)	D	-	*C*. *parvum*	IIaA15G1R1	*E*. *coli*
MH6	M	62	Nini (Tripoli)	AP/D/V/F	+	*C*. *hominis*	IdA19	-
MH7	F	7	Nini (Tripoli)	AP/D/V	+	*C*. *hominis*	IdA19	*E*. *histolytica*
MH8	M	15	Nini (Koura)	D/V/DH	+	*C*. *hominis*	IdA19	*E*. *coli*
MH9	M	2	Nini (Tripoli)	AP/D/V/F/DH	-	*C*. *hominis*	IdA19	-
MH10	M	1	Nini (Tripoli)	D/V/DH	-	*C*. *hominis*	IdA19	-
MH11	M	4	Nini (Tripoli)	D/V/F	-	*C*. *hominis*	IdA19	*E*. *coli*
MH12	M	2	Al-Youssef (Akkar)	D/V/F/DH	-	*C*. *hominis*	IdA19	-
MH13	M	1	Al-Youssef (Akkar)	D/V/F	-	*C*. *hominis*	IdA19	-
MH14	F	58	Al-Shifa’ (Tripoli)	AP/D	-	*C*. *hominis*	-	-
MH15	F	54	Nini (Koura)	AP/D/F	-	*C*. *hominis*	-	*E*. *histolytica*
MH16	M	13	Al-Shifa’ (Zgharta)	AP/D/V	+	-	-	-
MH17	F	2	Nini (Tripoli)	AP/D	+	-	-	-
MH18	F	1	Nini (Zgharta)	D/V/F	+	-	-	*E*. *coli*

M: Male, F: Female, AP: Abdominal pain, D: Diarrhea, V: Vomiting, F: Fever, DH: Dehydration, MZN: modified Ziehl-Neelsen

### Microscopic parasitological analyses

All fecal samples were examined macroscopically, and their characteristics, such as color, consistency, presence of blood, or presence of helminths were recorded. These specimens were also examined by direct-light microscopy of wet mounts. For the detection of *Cryptosporidium* spp. oocysts, modified Ziehl-Neelsen (MZN) staining was performed, and the slides were examined at 1000× magnification [19]. Two experienced microscopists observed all slides. No information was available on potential viral or bacterial infections in these fecal samples.

### DNA extraction, species identification and subtyping

All fecal specimens were used for molecular detection of *Cryptosporidium*. DNA was extracted from approximately 250 μg of fecal samples using the QIAmp DNA Stool Mini Kit (Qiagen GmbH, Hilden, Germany), according to the manufacturer’s recommended procedures. The DNA was eluted in 100 μl of elution buffer (Qiagen) and stored at −20°C until use. The 18S rRNA nested PCR was performed with primers, as previously described by Xiao *et al*. [20]. To test the potential presence of PCR inhibitors in fecal specimens, 0.5 μl of pure DNA of *C*. *parvum* was added to negative DNA samples and processed by PCR. To identify *Cryptosporidium* spp. molecularly, positive PCR products were purified and sequenced directly by the company Genoscreen (Pasteur Institute, Lille) on both strands using the forward and reverse primers used for the nested (secondary) PCR. The sequences obtained were aligned using the BioEdit v7.0.1 package (http://www.mbio.ncsu.edu/BioEdit/bioedit.html), then compared with sequences of *Cryptosporidium* published on the NCBI server (http://www.ncbi.nlm.nih.gov/BLAST/) using the basic local alignment search tool (BLAST) program. Specimens genotyped as *C*. *parvum* or *C*. *hominis* were further subtyped using a second nested PCR, which amplifies a fragment of the 60 kDa glycoprotein (gp60) gene, as described previously [21]. The amplified DNA fragments were purified, sequenced, and analyzed by alignment of gp60 sequences with reference sequences retrieved from GenBank using the program ClustalX (http://www.clustal.org/). *C*. *parvum* and *C*. *hominis* gp60 subtypes were named by counting the number of trinucleotide repeats of TCA (A), TCG (G), and TCT (T) and the ACATCA repeat (R) after the trinucleotide repeats [11]. The nucleotide sequences obtained in this study were deposited in GenBank under accession numbers KM215739 to KM215766.

## Results

A total of 163 stools from Lebanese hospitalized diarrhea patients were examined. The patients in this study came from different cities in northern Lebanon: 96 from Tripoli, 3 from Menyeh-Doniyeh, 5 from Koura, 10 from Zgharta, and 48 from Akkar. The male/female ratio was 0.9. Of the collected samples, 57.7% (94/163) were from children, and 42.3% (69/163) from adults. Besides diarrhea, patients could present other symptoms, such as abdominal pain (125/163), vomiting (34/163), and fever (21/163). *Cryptosporidium* spp. oocysts were found in 10 out of 163 (6.1%) samples examined by microscopy after MZN staining. After nested PCR analysis, *Cryptosporidium* spp. were detected in 15 samples (9.2%), and a total of 7 specimens out of these 15 samples were simultaneously positive by both methods. For the 3 samples that were positive by MZN staining but negative by nested PCR, the presence of inhibitors was confirmed using a PCR inhibition test. The overall prevalence of *Cryptosporidium* spp. infection obtained by MZN and/or nested PCR was 11% (Table 1), and children <5 years old showed a higher rate of *Cryptosporidium* spp. infection of 44.5% (8/18). The characteristic clinical data of *Cryptosporidium* spp. infected patients is shown in Table 1.

The PCR products of the 15 positive samples were successfully sequenced on both strands. The sequences obtained showed over 99% identity with the reference sequences. Among the isolates, 10 (66.7%) were identified as *C*. *hominis*, while the remaining 5 (33.3%) were identified as *C*. *parvum*. *Cryptosporidium* spp. other than *C*. *parvum* and *C*. *hominis* were not found.

The partial sequence of the gp60 gene was subsequently obtained for 8 *C*. *hominis* and 5 *C*. *parvum* isolates. Sequence analysis of the gp60 gene sequences identified all *C*. *hominis* isolates as belonging to the IdA19 subtype. Two subtypes of *C*. *parvum* belonging to the subtype family IIa were identified as follows: IIaA15G1R1 (4/5) and IIaA15G2R1 (1/5) (Table 1). The clinical manifestations associated with *Cryptosporidium* spp. infection are shown in Table 1.

Other intestinal parasites were detected by direct-light microscopy. In total, 79 out of 163 (48.5%) samples were positive for at least one intestinal parasitic infection. The distribution of these infections was as follows: *Blastocystis* spp. with the highest infection rate (19.6%), followed by *Entamoeba histolytica/dispar* (14.1%), *Entamoeba coli* (10.4%), *Giardia duodenalis* (3.7%), *Entamoeba hartmanni* (2.5%), *Taenia* spp. (0.6%), and *Trichomonas intestinalis* (0.6%). Overall, the prevalence of parasitic infections in males and females was 36.4% and 52.3%, respectively. Co-infection of *Cryptosporidium* spp. with other Protozoa was found in 7 cases (Table 1).

## Discussion

The present study represents the first report on molecular data of human cryptosporidiosis in Lebanon. Indeed, only very rare data are available in the literature regarding the situation of cryptosporidiosis in this Middle Eastern country, due to the lack of routine diagnosis of this parasite in medical laboratories. Although only one fecal specimen from each patient was analyzed in this study, thus decreasing the sensitivity of the diagnostic tools, it was shown that *Cryptosporidium* spp. infection was common among hospitalized Lebanese diarrhea patients, with a prevalence of 11%. No gender difference in the prevalence of the parasite was found, and children aged under 5 years were more frequently infected, as described previously [22]. Consistently, recent evidential data from the Global Enteric Multicenter Study (GEMS) on the burden and etiology of childhood diarrhea in developing countries has shown that *Cryptosporidium* spp. is nowadays a leading cause of moderate-to-severe diarrhea in children aged less than 2 years [23].

However, 6 (33%) immunocompetent adults were also infected in our cohort. Although cryptosporidiosis is less common among healthy adults, this observation has been reported previously [22]. The hospitalization of these adult patients due to the presence of gastrointestinal symptoms could explain the increase in *Cryptosporidium* detection due to a specific prescription of parasitological methods of diagnosis.

In our study, the frequency of the infection was higher in patients from Tripoli (61%) than from other cities in North Lebanon (Table 1). Globally, based on molecular tools for cryptosporidiosis detection among hospitalized patients, the prevalence of *Cryptosporidium* spp. in Lebanon (11%) was similar to that reported in Yemen (10%) [11], but lower than that reported in other neighboring countries, such as Jordan (19%) [12] and Egypt (49%) [13].

In our cohort of hospitalized patients, the molecular characterization of *Cryptosporidium* isolates identified *C*. *parvum* and *C*. *hominis*, with the latter being predominant. It is well known that human cryptosporidiosis is mainly caused by these two species, and that the distribution of *C*. *parvum* and *C*. *hominis* in humans differs in different geographic regions. In European countries, both *C*. *parvum* and *C*. *hominis* are common in humans [8]. In the Middle East, however, *C*. *parvum* is the dominant species in the human population [8]. In the rest of the world, especially in developing countries, *C*. *hominis* is usually the predominant species in humans [8], and the predominance of this species is related to anthroponotic transmission. Anthroponotic transmission of *Cryptosporidium* spp. has thus been described in several tropical countries, such as Peru, Thailand, Malawi, Uganda, Kenya, and South Africa [8].

In this study, all *C*. *hominis* isolates belonged to the Id subtype family, and were identified as belonging to the IdA19 subtype. Although the Id family is commonly reported around the world [8], the IdA19 subtype is less common. This subtype was previously described in Northern Ontario in Canada [24], and in hospitalized children in China [25]. In addition, patients with the IdA19 subtype presented severe symptoms (4 cases of dehydration and 7 cases of vomiting) in our report. Consistently, Iqbal *et al*. showed that the Id subtype family was associated with severe diarrhea lasting more than 6 days, and also associated with other clinical manifestations, such as fever and dehydration in Kuwaiti children [15]. This finding was also supported by an earlier study showing that the *C*. *hominis* Id subtype family was more virulent than other subtype families in Peruvian AIDS patients [26]. However, in the Chinese study mentioned above, IdA19 was not significantly associated with diarrhea [25].

On the other hand, two subtypes of *C*. *parvum* belonging to the IIa subtype family were identified in our study: IIaA15G1R1 (4/5) and IIaA15G2R1 (1/5). IIaA15G2R1 is the major subtype of *C*. *parvum* reported around the world, including in the Middle East and the Mediterranean Basin [8]. The *C*. *parvum* IIa subtype family, of which IIaA15G1R1 and IIaA15G2R1 are representatives, exhibits extensive genetic diversity and is responsible for the majority of cryptosporidiosis outbreaks due to *C*. *parvum* [8]. Moreover, this subtype family is dominant in the human population in the Middle East, with the exception of Jordan and Iran [11,12,15,16], and has been identified in both humans and animals. The IIc subtype family, associated with anthroponotic transmission, has been described in other Middle Eastern countries, but was not identified in our study [14,15].

Interestingly, the co-infection of *Cryptosporidium* spp. with pathogenic and nonpathogenic amoebas was found in several patients in our Lebanese cohort. Since amoebas are also transmitted by the fecal-oral route, these co-infections could indicate a common route of transmission for these parasites, probably due to contaminated food and water.

In conclusion, to our knowledge, this study is the first investigation regarding the molecular epidemiology of *Cryptosporidium* spp. in Lebanon. Our data indicates that the prevalence of cryptosporidiosis is relatively frequent among hospitalized diarrhea patients, in particular in children. Due to the limited number of isolates analyzed in this study, the epidemiologic significance of these results remains to be confirmed. For a better understanding of the circulation of this parasite and its transmission risk factors, further studies including a large number of human, animal, and environmental samples are needed.
